# The Potency of Goat Milk in Reducing the Induced Neurotoxic Effects of Valproic Acid in Rat Pups as a Rodent Model of Autism Spectrum Disorder

**DOI:** 10.3390/metabo13040497

**Published:** 2023-03-29

**Authors:** Alhanouf Mohammed Al-dossari, Laila Naif Al-Harbi, Norah M. Al-Otaibi, Abdullah Almubarak, Ahmed Tayseer Almnaizel, Ghedeir M. Alshammari, Ghalia Shamlan, Ali A. Alshatwi, Afaf El-Ansary

**Affiliations:** 1 Department of Food Science and Nutrition, College of Food Science and Agriculture, King Saud University, Riyadh 11451, Saudi Arabia; 2 Antimicrobial Resistance Division, Reference Laboratory for Microbiology, Executive Department of Reference Laboratories, Research and Laboratories Sector, Saudi Food and Drug Authority (SFDA), Riyadh 11671, Saudi Arabia; 3 Research Assistant, Veterinarian, Experimental Surgery and Animal Lab, Riyadh 12372, Saudi Arabia; 4 Experimental Surgery and Animal Lab, College of Medicine, King Saud University, Riyadh 12372, Saudi Arabia; 5 Central Research Laboratory, Female Center for Medical Studies and Scientific Section, King Saud University, Riyadh 11495, Saudi Arabia

**Keywords:** goat’s milk, cow’s milk, ASD, valproic acid, neurotransmitters

## Abstract

Autism spectrum disorder (ASD) is a progressively prevalent neurodevelopmental disorder with substantial clinical heterogeneity. Despite the considerable interest in dietary interventions, no consensus has been reached regarding the optimal nutritional therapy. The present study aimed to investigate the possible positive effect of goat’s milk (GM) compared to cow’s milk (CM) on ASD autistic features in a valproic acid (VPA; 600 mg/kg)-induced white albino rat model of autism. All tests were conducted on rats that were divided into four groups (*n* = 15/group): control with goat milk (GM) treatment, control with cow milk (CM) treatment, autistic with goat milk (GM) treatment, and autistic with cow milk treatment. The casein levels were also measured in GM and CM. Social behavior was assessed by three-chambered sociability to test social interaction after the intervention. After 15 days of intervention, selected biomarkers, such as glutathione (GSH), thiobarbituric acid reactive substance (TBARS), interleukin-6 (IL-6), neurotransmitter dopamine (DA), serotonin (5-hydroxytryptamine, 5-HT), and glutamate (GLU), were measured in blood serum and brain homogenates. The results showed a significant positive effect on social interaction in the VPA rat ASD model fed GM. Blood serum and brain samples showed a positive increase in TBARS in the VPA rat model fed GM, but brain and serum serotonin levels were lower in both VPA-GM and VPA-CM groups. Dopamine in serum was also lower in the VPA-CM group than in the VPA-GM group. IL-6 levels were slightly lower in the VPA-GM group than in the VPA-CM group. In comparison with cow’s milk, goat’s milk was effective in ameliorating the neurotoxic effects of VPA. Goat’s milk may be considered a suitable source of dairy for children diagnosed with ASD. Autistic children with allergies to cow’s milk could possibly convert to goat’s milk. Nevertheless, more in-depth studies and clinical trials are recommended.

## 1. Introduction

Autism spectrum disorder (ASD) is a progressively prevailing neurodevelopmental disorder with significant proven heterogeneity. As there are no cures for the disorder, treatments usually address the persistent behavioral and biochemical features of autism with the aim of improving the usual impaired social interaction and communication that those with ASD experience. While ASD is strongly influenced by genetics, environmental influences including toxins, pesticides, infection, and drugs are known to contribute as etiological factors. Specifically, exposure to valproic acid (VPA) during pregnancy has been shown to increase the risk of autism in children. Additionally, rodents prenatally exposed to VPA show behavioral and biochemical phenotype characteristics of the human condition [1]. VPA can alter the function of the neurotransmitter gamma amino butyrate (GABA) by potentializing the inhibitory functioning of GABA through many mechanisms [2]. A disturbed gut microbiota is a common comorbidity that is thought to be not only another symptom of ASD but also a target through which social and behavioral symptoms could be ameliorated. Therefore, nutritional interventions are used in the treatment of the majority of individuals with ASD, both with and without clinical supervision, to improve altered gut bacterial compositions. In spite of the considerable interest in dietary interventions, no consensus has been reached regarding the optimal nutritional therapy. Multiple nutritional interventions can be followed by those with ASD, such as adherence to gluten-free and casein-free, ketogenic, and specific carbohydrate diets, as well as the consumption of probiotics, polyunsaturated fatty acids, and dietary supplements (vitamins A, C, B6, and B12; magnesium and folate) [3]. Based on this, the concept of the brain–gut axis (GBA) has been extended to the microbiota–gut–brain axis (MGBA) [4].

Butts et al. [4] examined the effects of goat’s milk (GM) on re-establishing bacterial populations and metabolism in rats with amoxicillin-induced gut dysbiosis. Goat’s milk (GM) significantly increased the numbers of Bifidobacterium spp. and Lactobacillus spp. and decreased the numbers of *Clostridium perfringens* in the ceca and colons of rats treated with amoxicillin.

In numerous studies, bidirectional interaction between the GI tract and the brain has been reported. It was shown that communication takes place through the autonomic nervous system, the enteric nervous system, the neuroendocrine and neuroimmune systems, and the gut bacteria themselves, which modulate brain neurotransmitter production, affect brain development, and produce metabolites that greatly influence behavior in the rodent model of ASD [4,5].

It is interesting to know that the oligosaccharides in goat’s milk (GM) are more similar to those in human milk [6], with 4 and 10 times more fucosylated and sialylated oligosaccharides, respectively, compared to cow’s milk (CM) and sheep’s milk [6,7]. Based on the fact that the first two years of life represent an important period for brain development, not only due to the rapid proliferation and growth of neural cells, but also due to increases in synapse connections, sufficient early-life nutrition is essential to guarantee optimal cognitive development in infants and children [8]. Tarr et al. [8] reported that dietary supplementation with sufficient sialylated oligosaccharides (as an ingredient of goat’s milk) effectively improved colonic microbiota communities and reduced stressor-induced alterations in the intestinal mucosa structure, anxiety-like behavior, and immature neuron cell numbers in addition to their immune or endocrine effects. Goat’s milk has both acidic and neutral oligosaccharides, many of which are structurally comparable to human milk oligosaccharides (HMOs) [9,10,11,12]. Casein, as an animal protein found in cow’s, goat’s, sheep’s, and human milk, is not digested appropriately in people who have gut-related neurological disorders; its chemical structure turns into intermediate peptides and casomorphin, which has a similar effect to morphine. Casomorphin was found in urine samples taken from patients with autism [10]. These peptides cross the vascular structure through the blood to the brain and the fluid in the brain, causing the function of some areas of the brain to be hindered [11]. To reduce these symptoms and improve their experience with the disease, most people follow a gluten-free/casein-free (GF/CF) diet [11]. Research interest in goat’s milk is steadily increasing, with a primary focus on the physicochemical properties of A2 β-casein, as well as the easy digestibility and good hypoallergenic properties of goat’s milk in comparison to other types of milk [13,14]. Studies have shown that goat’s milk is very low in the allergic A1 β-casein and rich in A2 β-casein.

Therefore, the use of whole goat’s milk or specific fractions of goat’s milk may provide some prebiotic benefits for the development of a maturing gut, which can improve early brain development. To date, the effect of GM on ASD has not yet been investigated and remains unclear. Therefore, based on multiple previous studies which showed that the lower level of casein, the better brain development, and the much higher level of EPA and DHA in GM compared to CM which could help to reduce autistic features, it was hypothesized that GM may help to ameliorate biochemical and behavioral alterations in a VPA rodent model of ASD. The aim of the present study was to determine the effectiveness of goat’s milk—as a well-documented prebiotic with a high level of tolerable A2 β-casein—in amending persistent biochemical and behavioral features of autism. We examined this by giving goat’s milk to a valproic acid-induced rodent model of ASD.

## 2. Materials and Methods

### 2.1. Milk Products

The GM and CM products were obtained from a local organic market in Riyadh, Saudi Arabia.

### 2.2. Analysis of Fatty Acids in Cow and Goat Milk Products

The fatty acid profiles (saturated fatty acid, unsaturated fatty acid, monounsaturated fatty acid, polyunsaturated fatty acid, trans fatty acid, omega-3 precursor: linoleic acid, omega-6 precursor: A-linolenic) were analyzed in GM and CM using fatty acid methylation kits via gas chromatography with flame-ionization detection (GC-FID) [15], as per AOAC-996.01.

### 2.3. Quantification of Milk Casein Content

The casein content in GM and CM was quantified according to the method of Kumaresan [16,17,18,19]. Samples (60 mL) of GM and CM obtained from a local market were heated to 50 °C in a 140 mL beaker; then, 14 mL of 6% acetic acid was added gradually while being stirred until the pH reached 4.6. The milk solution was left undisturbed for 10 min, and then, it was filtered through a muslin cloth to collect the casein precipitate and washed with distilled water to remove excess acid. The casein precipitate was insoluble in H_2_O, alcohol, and ether but dissolved in alkaline solutions (Applied Biochemistry Lab. BIOC 44). The casein molecules precipitated at the bottom of the beaker and were repeatedly filtered through the muslin cloth before the fat was removed using 20 mL of 96% ethanol. The precipitate was filtered through Whatman filter paper and air-dried in a Petri dish for 24 h. The casein was weighed accurately to the gram to calculate the percentage.

### 2.4. Experimental Design

All the experimental procedures were approved by The Research Ethics Committee (REC) at King Saud University, Ethics Reference No. KSU-SE- 20-28 on 14/5/2020 G, 21/9/1441 H. Eight female Wistar albino rats (150–160 g) were housed in air-conditioned standard cages at 21–23 °C and 60–65% relative humidity with 12 h light/12 h darkness and fed a standard laboratory diet and water ad libitum. The fertility cycles of all of the rats were monitored via daily vaginal smears. When the rats were confirmed to be fertile, the female rats were put in a cage with one male overnight. Vaginal smears were also used to determine if pregnancy had occurred. The first day of the gestational period is considered the day when the protein coagulates were observed in the vaginal smear. After the pregnancies of eight female rats were confirmed, the rats were weighed on day 12.5 of gestation, and all of the rats weighed 299–300 g. The pregnant rats were divided into two sets: set I was injected with a single intraperitoneal injection of 0.9% normal saline (1.5 mL)—the rat pups born from these female rats were the control group rats—and set II was injected with a single intraperitoneal injection of 600 mg/kg VPA according to the method of Al-Askar et al. [19]. Females treated with valproate (VPA) and control (C) were permitted to rear their litters. A twice-daily checkup for the presence of new litter was performed. When the litter was initially sighted, we assigned it a postnatal day (PND) of zero.

In the home cage, the mother and new litter were kept together. Male neonatal rats from both sets were differentiated by observing the urinary papilla and genital opening on PND 4.

After birth, the male pups were kept with their mother until they were weaned on PND 21–35 due to the differences in their weight. The body weight gains of rats were measured before and after the intervention. On postnatal day (PND) 21–35, the VPA-treated and the control pups (30–50 g) were divided into four groups (15 rats/group) and supplemented with either GM or CM. Group I: non-VPA pups were orally supplemented with GM; Group II: non-VPA pups were orally supplemented with CM; Group III: VPA pups were orally supplemented with GM; Group IV: VPA pups were orally supplemented with CM. All of the rats were fed via oral gavage (2 mL/rat) twice daily at 9 am and 4 pm for 15 days [15]. A control group that underwent no treatment was not included due to the validation of VPA’s negative effect on rats through past studies [20].

### 2.5. Three-Chamber Sociability Test (TCSA)

The three-chamber sociability test (TCSA) was used to track social approach behaviors related to autism, and an intervention was performed to validate their autistic features and to compare the differences. The three-chamber test measures general sociability and interest in social novelty in rodent models of CNS diseases to evaluate cognition. The three-chamber test can help identify rats that have impaired social interaction based on these inclinations. Three sessions of testing are conducted in a three-chambered box with openings between the chambers. After becoming accustomed to the three-room arena, the subject rat is placed in the central chamber. In the left chamber of the testing apparatus, a stimulus rat is placed inside a wire cage. The right room contains a similar wire cage without the stimulus rat [21]. The duration of time the experimental rat spends in each room and the amount of time it spends smelling each wire cage are both tracked to evaluate the animal’s sociability. Normal rats will spend more time with the conspecific than with the empty room, whereas rats with impaired social interaction will spend more time with the empty chamber than with the conspecific [21].

### 2.6. Biochemical Assays in Brain Tissue and Serum

At the end of 15 days of the treatment period, serum and brain homogenate were placed in phosphate buffer (pH 7.4) to yield 10× (*w*/*v*). All samples were collected and stored at −80° for biochemical assays.

Lipid peroxidase was measured using the OXItek TBARS Assay Kit (Cat. No.: 0801192, Main Street Buffalo, New York, NY, USA).

The Cayman GSH Assay Kit (Cat. No.: 703002, Ellsworth Rd, Ann Arbor, MI, USA) was used to quantify the GSH levels in the serum and brain samples.

IL-6 was measured using a competitive ELISA kit (Cat. No.: MBS726707, My BioSource company, San Diego, CA, USA) according to the manufacturer’s instructions.

Dopamine (DA) levels were measured using a competitive ELISA kit (Cat. No.: MBS725908, My BioSource company, USA) according to the manufacturer’s protocol.

Serotonin (5-HT) levels were measured using a competitive ELISA kit (Cat. No.: MBS725497, My BioSource company, USA) as per the manufacturer’s instructions.

Glutamate (GLU) levels were measured using a competitive ELISA kit (Cat. No.: MBS756-400, My BioSource company, USA) according to the manufacturer’s protocol.

Total protein (color/endpoint) was measured in serum via the biuret reaction according to the method described by Buzanovskii [22].

### 2.7. Statistical Analysis

The biochemical results are presented as mean ± SD (standard deviation). One-way analysis of variance (ANOVA) and Tukey’s multiple comparisons were performed between the measured variables using GraphPad Prism 8.4.0. A *p*-value ≤ 0.05 was considered statistically significant. The behavior data presented as interaction behavior and locomotion activity were compared using one-way ANOVA. * *p* < 0/05 bars, which were means ± SD, were created using IBM SPSS in the before and after treatment data, and Tukey’s multiple comparisons were performed. Furthermore, a *t* test was performed on the chemical analysis of the milk. The gains in body weight were expressed as mean ± stander error of the mean (SEM). The initial and final weight gains were measured using one-way ANOVA followed by the Newman–Keuls multiple comparison test.

## 3. Results

### 3.1. Milk Chemical Analysis

Fatty acid profile analyses included the ω-3 and ω-6 precursors, in both CM and GM, to confirm their levels and compare them via gas chromatography flame-ionization detection. As shown in Table 1, the profile of goat’s milk displayed a significantly higher fatty acid content than cow’s milk. Moreover, the ratios of the ω-3 and ω-6 precursors were found to be much higher in goat’s milk.

As shown in Table 2, the Quantitative analysis of casein levels in goat’s and cow’s milk.

As shown in Table 3, the increases in the rats’ body weights were statistically significant, as shown in the comparison of the final weights and the body weight gain in the control goat’s milk condition and the VPA goat’s milk condition.

### 3.2. Analysis of Social Behavior

**Figure 1:**
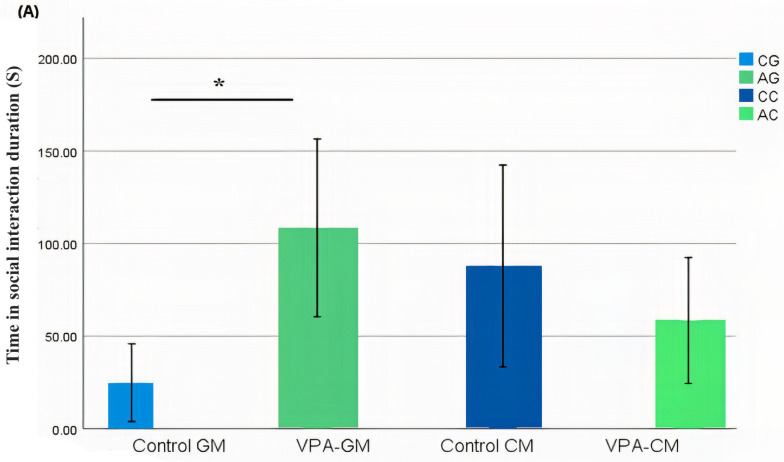

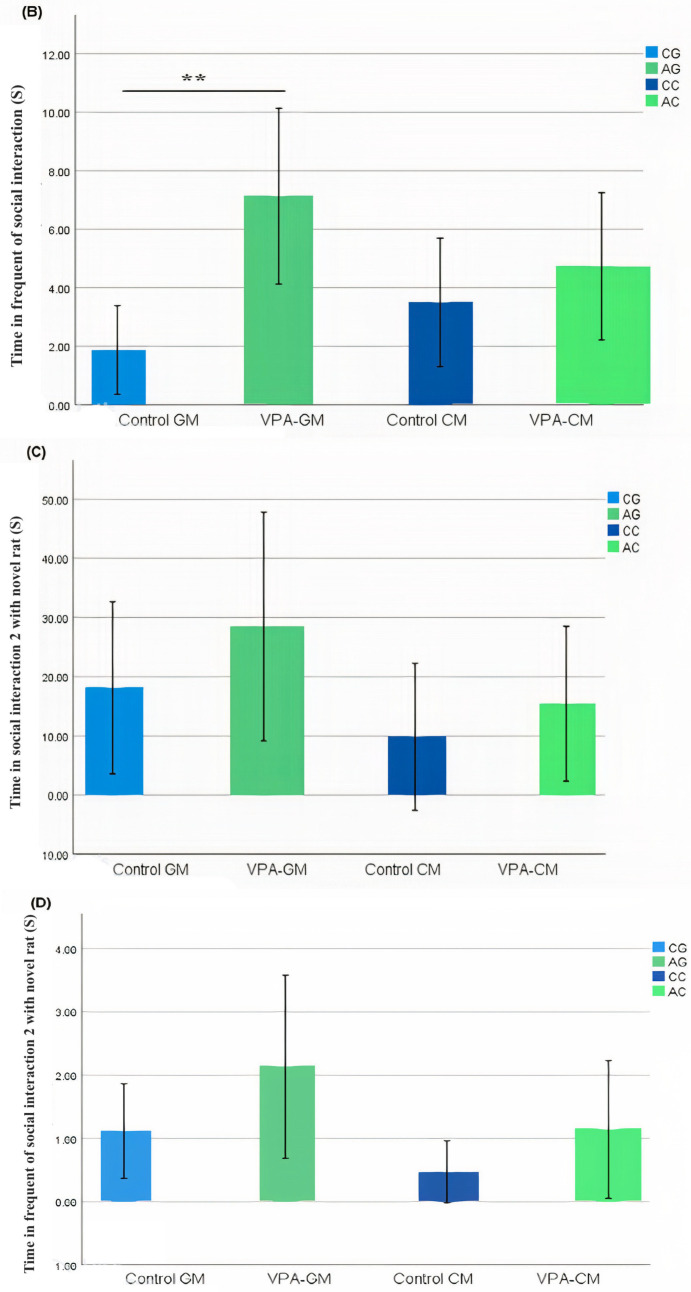

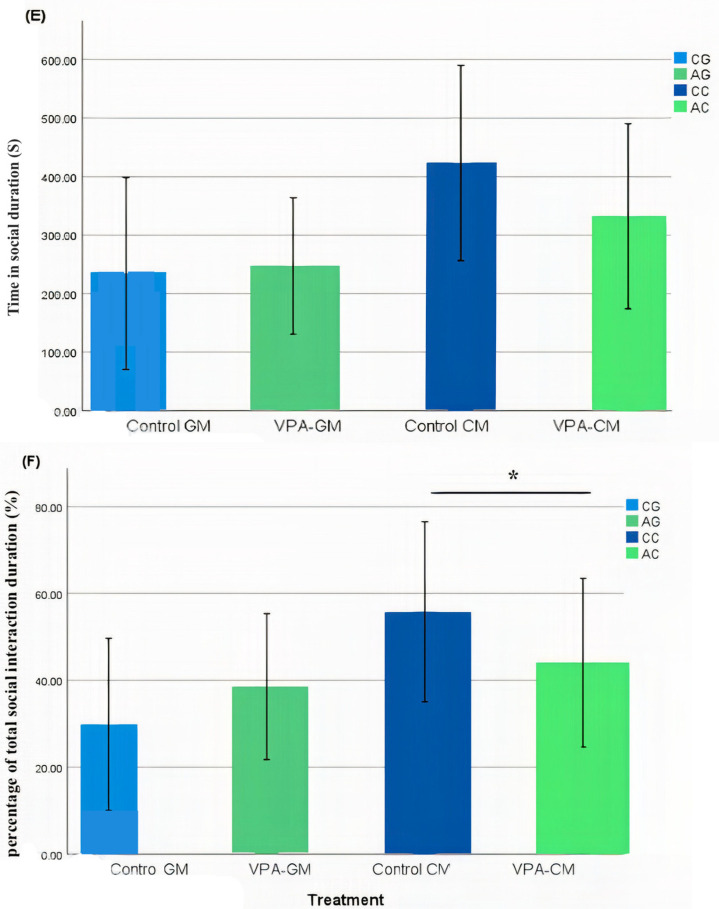
(**A**–**F**) Analysis of all behavioral phenotypes after the intervention. Additionally, (**A**) shows the duration spent in the social chamber. The control cow (CC) (n = 19), control goat (CG) (n = 16), autistic cow (AC) (n = 13), and autistic goat (AG) (n = 15) groups are shown. The bars are mean ± SEM. The data were analyzed via a two-way ANOVA (* *p* < 0.05). (**A**) There were significant differences between the control GM and VPA-GM (*p =* 0.014) groups. Additionally, in the VPA-GM group, there was a remarkable increase in the time spent socially interacting compared to rats fed CM. Furthermore, (**B**) displays the CC (n = 18), CG (n = 16), AC (n = 15), and AG (n = 15) groups. The bars are mean ± SEM. The data were analyzed with a two-way ANOVA (* *p* < 0.05). (**B**) There was a significant difference between the control GM and VPA-GM groups. Additionally, in the VPA-GM group, there was a remarkable increase in the frequency of social interaction compared to rats fed CM. Additionally, (**C**) displays the CC (n = 17), CG (n = 16), VPA-CM (n = 15), and VPA-GM (n = 15) groups. The bars are mean ± SEM. The data were analyzed using a two-way ANOVA (* *p* < 0.05). (**C**) There was no significant difference in the time spent socially interacting with the novel rat zone. However, in the VPA rats fed GM, there was a remarkable increase in interaction with the novel rat zone compared to the control rats fed GM and the VPA-CM group. Additionally, in (**D**), the CC (n = 17), CG (n = 17), AC (n = 14), and AG (n = 15) groups are displayed. The bars are mean ± SEM. The data were analyzed via a two-way ANOVA (* *p* < 0.05). (**D**) There was no significant difference between the groups. However, in the VPA rats fed GM, a remarkable increase in the frequency of social interaction with novel rats was shown compared to the control GM and VPA-CM groups. Moreover, (**E**) displays the CC (n = 19), CG (n = 17), AC (n = 15), and AG (n = 15) groups. The bars are mean ± SEM. The data were analyzed via a two-way ANOVA (* *p* < 0.05). (**E**) There was no significant difference between the groups. However, the control groups fed GM and CM displayed a slight increase compared to VPA rats fed GM and CM. Additionally, (**F**) displays the CC (n = 19), CG (n = 17), AC (n = 15), and AG (n = 15) groups. The bars are mean ± SEM. The data were analyzed with a two-way ANOVA (* *p* < 0.05). (**F**) There was a significant difference between the control CM and VPA-CM groups (*p* = 0.0369). However, the percentage in VPA rats fed GM was remarkably higher than that in control rats fed GM, while in the control CM group, the percentage was much higher than that in the VPA-CM group. *p*-value was considered significant when * *p* < 0.05 ** *p* < 0.01).

In the current study, we determined whether behavioral phenotypic modifications took place using the three-chamber social test. All of the rats, which were either administered a single intraperitoneal injection with valproic acid or not, were examined after the intervention. Data coding of the social behavior of the rats was measured using BORIS software by targeting the time spent socially interacting (Figure 1A), the frequency of social interaction (Figure 1B), the time spent socially interacting with novel rats (Figure 1C), the frequency of social interaction with novel rats (Figure 1D), the time spent socializing from the start to the end of the test (Figure 1E), and the percentage of total social duration (Figure 1F).

### 3.3. Analysis of Biochemical Variables

**Figure 2:**
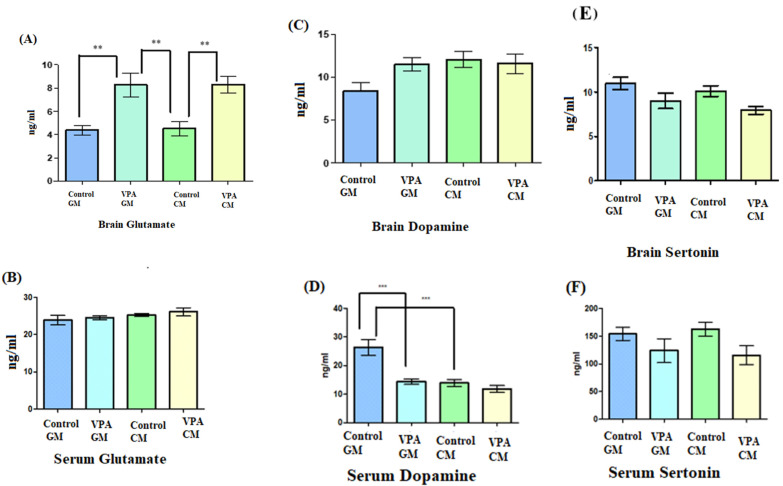
(**A**–**F**) Levels of neurotransmitters in rat serum and brain. GM- and CM-treated VPA rats in a rodent model of autism; data shown as mean ± SD. Data analysis was carried out via one-way ANOVA followed by Tukey’s post-comparison test; *p*-value was considered significant when *p* (** *p* < 0.01, *** *p* < 0.001).

The data shown in Figure 2A demonstrate a significant increase in the glutamate levels in the brain in the VPA rodent model of autism fed either GM or CM compared to the control. Moreover, no significant difference was observed in glutamate levels in the VPA rodent ASD model between the rats fed GM and CM. Figure 2B demonstrates that the glutamate levels in serum were not significantly different, and the goat’s milk treatment did not affect the VPA-GM group. Moreover, the glutamate level in the VPA-CM group was slightly higher than that in the VPA-GM group. Furthermore, as shown in Figure 2C, while the levels of dopamine in the brain did not show any significant differences between the groups, Figure 2D shows that the level of dopamine in the serum displayed significant differences (*p* < 0.0001), with a lower level of dopamine being recorded in the VPA-CM group than in the VPA-GM group. Furthermore, as shown in Figure 2E,F, the levels of serotonin in the brain and serum were lower in both the VPA-GM and VPA-CM groups.

**Figure 3:**
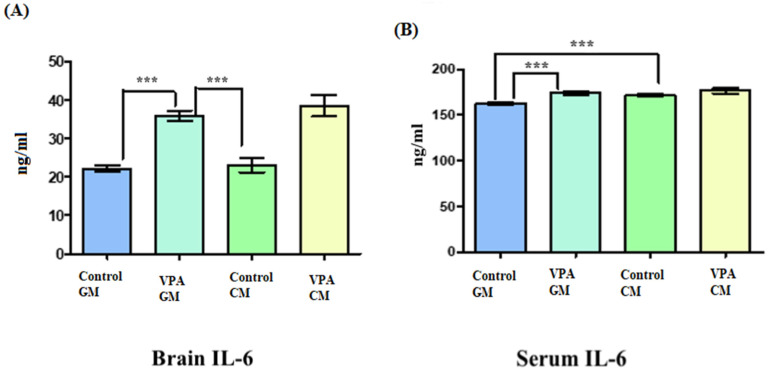
(**A**,**B**) Interleukin-6 proinflammatory markers in rat serum and brain. GM- and CM-treated rats shown by mean ± SD. Data analysis was carried out via one-way ANOVA followed by Tukey’s post-comparison test; *p*-value was considered significant when *p* < 0.05. (**A**) Interleukin-6 level in brain tissue. (**B**) Interleukin-6 level in blood serum (*** *p* < 0.001).

Figure 3A shows IL-6 in brain tissue, and Figure 3B shows IL-6 in serum. In Figure 3A, the IL-6 level in the brain tissue was slightly elevated in the VPA rodent ASD model fed CM compared to the VPA rodent ASD model fed GM. Additionally, there were significant differences between the brains of the control GM and VPA-GM rats, as well as significant differences between the VPA-GM and control CM groups (*p* < 0.0001). (B) There was a slightly increased level of IL-6 in the VPA-CM group compared to the VPA-GM group. Additionally, the level of IL-6 in the serum was significantly different between the control GM and VPA-GM groups (*p* < 0.0001).

**Figure 4:**
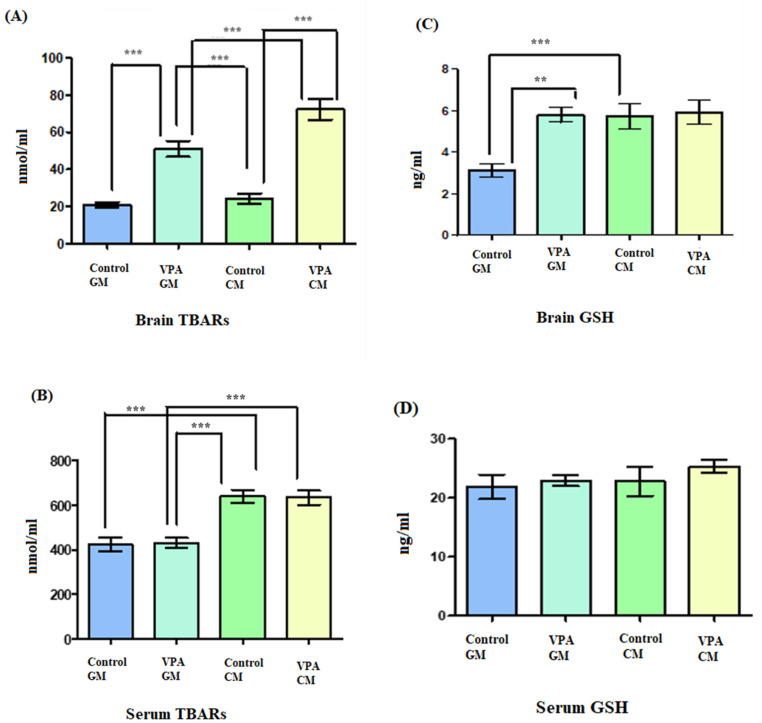
(**A**–**D**) Oxidative stress markers in rat serum and brain. GM- and CM-treated rats shown by mean ± SD. Data analysis was carried out via one-way ANOVA followed by Tukey’s post-comparison test; *p*-value was considered significant when *p* < 0.05 (** *p* < 0.01, *** *p* < 0.001).

Figure 4A shows TBARS lipid peroxide in brain tissue, and Figure 4B shows TBARS lipid peroxide in serum. In Figure 4A, a significant increase in lipid peroxides is demonstrated as a marker of oxidative stress in both the VPA rodents and in the controls. Additionally, there was a significant difference between the VPA rats fed goat’s milk and the control rats fed goat’s milk (*p* < 0.0001), as well as a significant difference between control rats fed cow’s milk and the VPA rats fed cow’s milk (*p* < 0.0001) (Figure 4B). In the control CM group, a significantly higher level of lipid peroxidation was recorded compared to that in the control GM group. Moreover, there was a significant difference in the serum between the VPA rats fed cow’s milk and the VPA rats fed goat’s milk (*p* < 0.0001).

Additionally, in Figure 4C, the glutathione level in brain tissue is shown; in Figure 4D, the glutathione level in serum is shown. In Figure 4C, showing the VPA rodent ASD model fed GM, it can be seen that the brain tissue displayed increased levels of glutathione compared to the control GM group. Furthermore, there was a significant difference in the GSH levels in the brain between the VPA-GM and control GM groups (*p* = 0.0004).

## 4. Discussion

Based on the fact that a cow’s milk allergy (CMA) is considered the most common food allergy in the early life of infants, goat’s milk has emerged as a substitute that can be easily digested [23]. Of all allergy proteins, caseins account for approximately 80% of the total protein and are considered to be the major allergy protein group in cow’s milk. Among the four subgroups of caseins (α_S1_-, α_S2_-, β-, and κ-CN) in cow’s milk, α_S1_-CN was reported to have the strongest allergenic activity. Table 1 demonstrates that goat’s milk contains a lower amount of α_S1_-CN. This supports previous studies that claimed goat’s milk formula has significantly lower As1-casein levels than cow’s milk formula, and its protein profile is more in line with the casein profile of human milk [24].

The results of the current study’s analysis of fatty acid profiles, which included the -3 and -6 precursors, showed noticeably high levels of these precursors in GM. This is in line with the conclusions of earlier studies that “goat milk exceeds cow milk in monounsaturated, polyunsaturated fatty acids, and medium-chain triglycerides.” [25] (Table 2). This is supported by Idamokoro et al. [26], who reported that goat’s milk is an important source of PUFAs as health-promoting substances and suggest the sustainable use of animal genetic resources to improve the fat quality of goat’s milk so that it provides important nutritional value related to normal brain development and the function of nervous tissue through the critical role of ω-3 FAs such as EPA and DHA and their precursors in neurotransmission, neurogenesis, and protection from oxidative stress [27]. The physiological and biochemical effects and properties of GM are scarcely known and have been minimally exploited, and thus, further research should be encouraged.

Furthermore, the effect of VPA neurotoxicity on social interaction and behavior was investigated after the intervention (Figure 1A–F). Tartaglione et al. [28] provided evidence—in agreement with clinical evidence—that prenatally exposing rodents to VPA will cause them to display persistent behavioral features of autism. Our non-significantly different behavioral data could be explained on the basis that it is recommended to start behavioral testing in rats during the adolescence period, which begins 63 days after birth and follows the eighth week of postnatal life, due to an increase in social play and risk-taking during this time [29]. Moreover, in a systematic review study, it was interestingly observed that there was a decreased level of social interaction in older age groups of VPA rats; these age groups also had a lower discrimination index in the novel object recognition test compared to younger VPA rodents. It was concluded that VPA studies using adolescent rodents are more optimal due to their clear display of social impairment [30]. Another study contradicts Chaliha; it was reported that the postnatal exposure of rats to VPA leads to autistic-like behavior in rats due to an increased number of astrocytes in the frontal cortex, which may alter the establishment of appropriate neuron–glial circuit formation, which affects functional deficits. Astrocytes are known to play an important role in neural circuit development. The three-chamber social test was performed at PND 21 or 22, which revealed that the rats experienced social interaction deficits [31].

Additionally, the results of the behavior interaction test following the nutritional intervention period revealed a significantly better outcome in the GM treatment group for the autistic rodent model compared to the GM treatment group for controls, as well as a marginally better result in the GM treatment group compared to the CM treatment group (Figure 1). This can be explained by the work of [32,33], in which it was shown that the consumption of goat’s milk increased dopamine, oxytocin, serotonin, synaptophysin, and α-MSH, which are neurotransmitters and neuropeptides related to ASD, suggesting a potential neuroprotective effect in brain functions, which could enhance social interaction behavior [34,35,36,37].

As a marker of excitotoxicity, our results showed that the glutamate levels were significantly different between the CC, AC, CG, and AG groups; however, no significant positive effect was shown for GM treatment (Figure 2A,B). This could help to suggest that glutamate excitotoxicity is a causal mechanism in both ASD patients and the VPA animal model [33,38,39]. This suggestion can find great support in the recent finding of El-Ansary [39] indicating that imbalanced glutamate/GABA or excitatory/inhibitory neurotransmission is accepted as a neurochemical etiological mechanism of ASD. Dopamine levels have been associated with learning, memory, and attention as well as various hormonal pathways, stress responses, addiction, and emotional behavior [40]. The treatment with goat’s milk showed a remarkable increase in the dopamine levels in the brain homogenate in VPA GM-treated rats, concomitant with a much lower level in serum in the VPA-GM group (Figure 2C,D). This could be attributed to the high level of FAs and omega-3 in goat’s milk, which have essential roles in reducing stress, depression, and dementia [41]; GM also directly increases dopamine levels [40].

Figure 2E,F demonstrate the non-significant change in the level of serotonin in the brain and serum in the four studied groups, which contradicts the suggestion that hyperserotonemia is one of the most commonly observed changes in VPA rodent models of autism [42]. This discrepancy could be attributed to the age of the rat pups employed in the current investigation, which was younger than the recommended age for inducing some autistic characteristics [43].

The sensitization of α_S1_-CN in cow’s milk mostly presents itself as damaged intestinal barriers in mice, the leakage of LPS, the activation of the TLR4-NFκB pathway, and thus, the increase in the level of proinflammatory cytokines, which triggers systemic inflammation [24]. Figure 3A,B demonstrate slightly elevated levels of IL-6 in the VPA rodent ASD model fed CM compared with the VPA rodent ASD model fed GM, which could, to a certain extent, support the sensitization of the VPA-ASD rodent model to the higher α_S1_-CN level in CM compared with GM (4% versus 3.2%) [24]. This is in good agreement with the study by Sunarti et al. [32], who showed that GM and soybean milk kefir can help reduce the level of IL-6 due to the composition of GM, which contains oligosaccharides that help reduce the level of inflammation in the intestine and colon.

Furthermore, oxidative stress and imbalanced neurotransmitters have been established as biochemical markers in ASD patients. While the levels of TBARS, glutamate, and IL-6 are much higher in ASD patients, usually, glutathione, serotonin, and dopamine levels are lower compared to healthy controls in ASD patients and rodent models [20,44,45,46].

Figure 4A,B present the significantly higher level of TBARS in serum in VPA CM-treated rats than in VPA GM-treated rats, which could be elucidated via leaky gut syndrome, which is frequently observed in autistic patients. The presence of a higher level of TBARS can be explained by the higher levels of A1 casein in CM compared to GM [47,48].

Furthermore, convincing evidence connects A1β-casein and mental disorders, due to the increased permeability associated with digestive disorders. In the case of leaky gut, free radicals are present in considerably higher concentrations in the brain and blood than in those with a healthy gut wall that does not leak [49]. In the current study, the level of TBARS, as an oxidative stress biomarker, was much lower in VPA GM-treated rats in both brain homogenate and serum, which supports our hypothesis and agrees with the results of previous studies regarding the possible role of the microbiota–gut–brain axis in autism spectrum disorder [50,51] and that healthy food is usually allied with a healthy gut. In contrast, in our study, no differences in the levels of GSH in VPA GM- and CM-treated brain samples were demonstrated (Figure 4C,D). These results do not support our hypothesis, but the comparison of the GM treatment to the CM treatment did demonstrate a slight increase. This can be explained by the gut microbiota community and diversity in ASD. In ASD, the gut microbiota modulates and dysregulates the metabolism of GSH [45,52]. The effect of goat’s milk on GSH levels could be attributed to its prebiotic effects, which manifest as an increased abundance of Bifidobacterium spp. and Lactobacillus spp. as probiotics and a decreased abundance of *Clostridium perfringens* as bacterial species known to be dramatically altered in autistic patients and rodent models [37,53,54,55].

## 5. Conclusions

In conclusion, the result of the present study states that VPA intoxication can lead to disruptions in brain metabolism through the remarkable increase in oxidative stress, neuroinflammation, and glutamate toxicity as biochemical autistic features. Amelioration of VPA-induced neurotoxicity can be attained through the consumption of goat milk. Thus, sufficient dietary intake of goat milk by individuals at high risk of VPA exposure could prove beneficial in combating the adverse effects of VPA. However, some more detailed and in vivo studies are necessary.

## 6. Limitation

The variability of the animals’ weaning age is a significant drawback of the current study, as the progressive changes in the mother–infant bond throughout weaning have significant relevance within the framework of development.

## Figures and Tables

**Table 1:** Fatty acid, ω-3, and ω-6 precursor profiles determined via GC-FID.

Fatty Acid Profile	Goat’s Milk	Cow’s Milk	*p*-Value
Saturated Fatty Acid	2.80 g/100 g	1.96 g/100 g	0.8288
Unsaturated Fatty Acid	1.24 g/100 g	1.03 g/100 g	0.9083
Monounsaturated Fatty Acid	0.91 g/100 g	0.82 g/100 g	0.9481
Polyunsaturated Fatty Acid	0.19 g/100 g	0.14 g/100 g	0.8518
Trans Fatty Acid	0.14 g/100 g	0.07 g/100 g	0.6985
C18:2n6c (Linoleic)	3.81%	3.69%	0.984
C18:3n3 (A-Linolenic)	0.57%	0.49%	0.925
Ratio of ω-3 and ω-6 precursors	1.27:0.19	0.163333:1.23	

**Table 2:** Quantitative analysis of casein levels in goat’s and cow’s milk.

Name	Temperature	PH	ML	Casein g	Casein%	*p*-Value
Goat’s milk	50 °C	4.6	60	1.95 g	3.2%	0.8850
Cow’s milk	50 °C	4.6	60	2.43 g	4.0%	

**Table 3:** Body weight gains in rats.

Groups	Initial Weight (g)	Final Weight (g)	Body Weight Gain (g)
Control Cow	35.67 ± 1.116	84.47 ± 3.022	48.80 ± 2.698
VPA Cow	37.27 ± 1.080 *	94.60 ± 4.022	57.33 ± 3.915
Control Goat	36.07 ± 0.9333 *	67.27 ± 3.087 ***	31.20 ± 2.675 ***
VPA Goat	41.07 ± 1.672 *	95.53 ± 3.290 ***	54.47 ± 2.635 ***

Values are expressed as means ± standard error of the mean (SEM) of 15 rats treated for 15 days. * *p* < 0.0120 and *** *p* < 0.0001. Determined using one-way ANOVA followed by Newman–Keuls multiple comparison test.

## Data Availability

The datasets generated during and/or analyzed during the current study are available from the corresponding author on reasonable request.
